# Patterns and Grade of Velopharyngeal Closure in Candidates for Adenotonsillectomy

**Published:** 2018-01

**Authors:** Mohammad-Waheed El-Anwar, Ezzeddin El-Sheikh, Nasser El-Nakeb

**Affiliations:** 1 *Department of Otorhinolaryngology-Head and Neck Surgery, Faculty of Medicine, Zagazig University, Zagazig, Egypt.*

**Keywords:** Adenotonsillectomy, Speech assessment, Velopharyngeal valve, Video-nasoendoscopy

## Abstract

**Introduction:**

The most common type of velopharyngeal valve (VPV) closure is a matter of controversy. The aim of this study was to estimate the most common type of VPV closure, including identification of the type and grade of VPV closure among Egyptian children.

**Materials and Methods:**

This study included patients who were candidates for adenotonsillectomy. In these cases, video-nasoendoscopy and speech assessment were performed prior to surgery in addition to otorhinolaryngology examination and preoperative laboratory tests.

**Results:**

By examination of 97 patients, video-nasoendoscopy of the VPV revealed that 49 patients had coronal VPV closure (50.5%), 48 patients had circular VPV closure (49.5%) and no patients had sagittal VPV closure.

**Conclusion:**

Coronal and circular closure are the main types of VPV closure in Arabic-speaking children, as neither sagittal closure nor circular closure with Passavant’s ridge were detected in this study. Therefore, revision of the methods of repair for persistent velopharyngeal insufficiency (VPI) is required.

## Introduction

The velopharyngeal valve (VPV) is a muscular valve that separates the nasopharynx from the oropharynx. It is formed by the velum (soft palate) anteriorly, lateral pharyngeal walls (from both sides) and posteriorly to the posterior pharyngeal wall. The main function of the VPV is to create a tight seal between the velum and the pharyngeal walls, separating the oral cavity from the nasal cavity for both pneumatic purposes during speech and non-pneumatic purposes, such as during swallowing (1).

The proper articulation of speech requires an intact dynamic interaction between the palatal muscles and the pharyngeal wall muscles, which is adjusted by the neural pathway that controls the VPV. As the larynx is a resonating cavity, in order to produce various sounds and maintain the intelligibility of speech, sound coupling and uncoupling between the oral and nasal cavities is needed. The main VPV function is to control the acoustic energy and airflow under different pressures through the nasal and oral cavities. In the production of nasal sounds (m,n,ng), the VPV permits transmission of air through the nasal cavity, while for oral sounds (all vowels and remaining consonants), the VPV seals off, allowing air only to pass through the oral cavity (2).

There are various patterns of VPV closure, with three basic patterns having been described in normal subjects; coronal, circular (with or without Passavant’s ridge) and sagittal (3,4). However, the most common type of VPV closure, either coronal or circular, remains a matter of controversy (5,6).

The aim of this study was to identify of the patterns and grade of VPV closure in Arabic-speaking children and to predict the patients who could have complications such as open nasality or nasal regurgitation after adenotonsillectomy.

## Materials and Methods

This study was conducted in the Department of Otorhinolaryngology and Phoniatrics Unit, Zagazig University Hospital between November 2014 and March 2016. The study was approved by the Institutional Review Board (IRB). The study included Arabic-speaking patients with normal speech who were scheduled for adenotonsillectomy. Patients with cleft palate, submucous cleft palate, or history of cranio-maxillo-facial abnormality, injury or surgery was excluded from the study. All patients were subjected to a full history, general, local and phoniatric examination, and routine preoperative laboratory tests. Then, a flexible nasofibros- copic examination and recording of the VPV closure type and grade were performed.

*Video-nasoendoscopy*

All patients were examined using a flexible fiberoptic nasopharyngoscope (Xion Medical, Berlin, Germany). The VPV movement was recorded during swallowing then while the patient was repeating the speech samples following a recommendation given by an international working group (7).

The following patterns of VPV closure were described: (1) coronal, in which velar elevation is the dominant movement for the VPV closure; (2) circular (with or without Passavant’s ridge), in which there is a near equal movement of the velum anteriorly and the lateral pharyngeal walls on both sides during the VPV closure and (3) sagittal, in which medial movement of the lateral pharyngeal walls is the dominant movement for VPV closure (3,4). The movement of the velum and lateral pharyngeal walls was traced on the monitor and given a score from 0 to 4 as follows: 0=resting (breathing) position or no movement; 2=half the distance to the corresponding wall; 4= maximum movement reaching and touching the opposite wall (8).

The movement was recorded, then the VPV closure was reevaluated and judged later by other researchers who were blinded to evaluation of each other.

*Statistical analysis*

Data were collected, tabulated, and analyzed. Correlations between patterns of closure and demographic patient data were calculated. Statistical analysis was performed using the software package SPSS (version 10.0 for Windows; SPSS Inc., Chicago, I ll). The chi-square test was used for qualitative variables, and the paired T-test was used for quantitative variables. A p-value less than 0.05 was considered to be significant.

## Results

**Fig 1:**
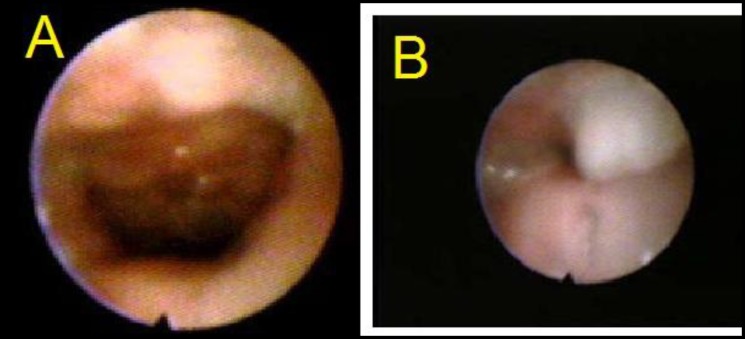
Velopharyngeal valve (VPV) at rest (A) and at speech (B) showed circular closure of Grade 4

**Fig 2:**
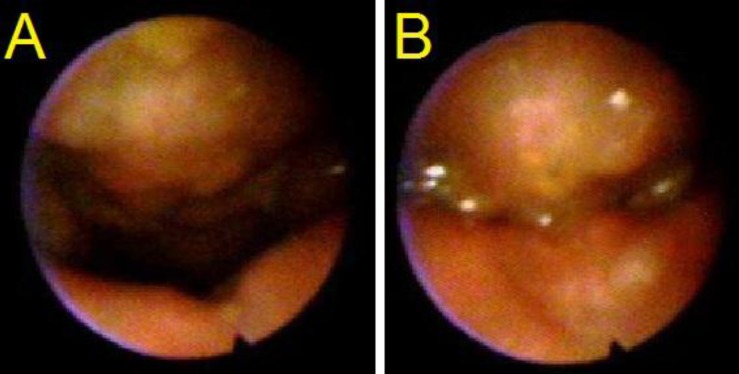
VPV at rest (A) and at speech (B) showed coronal closure of Grade 4

**Fig 3:**
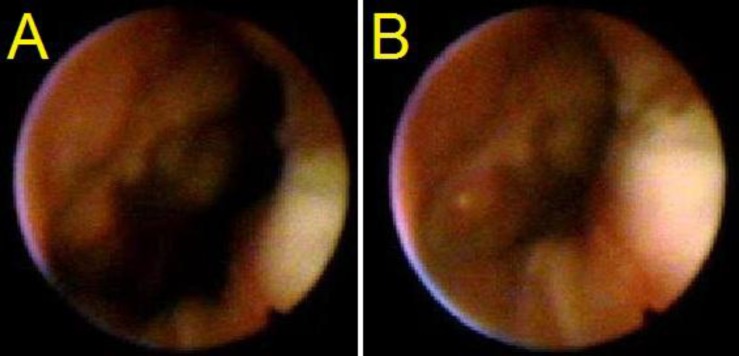
VPV at rest (A) and at speech (B) showed coronal closure of Grade 3

**Fig 4:**
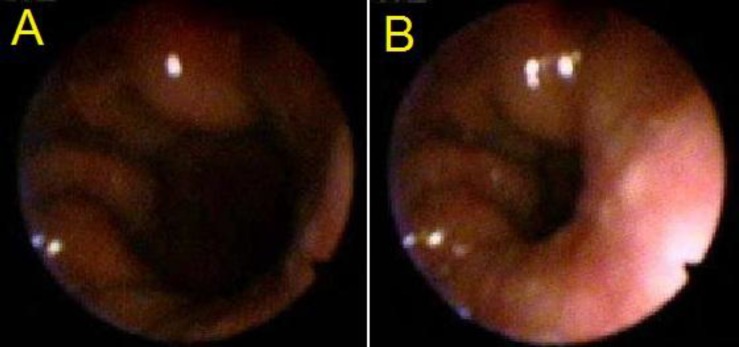
VPV at rest (A) and at speech (B) showed circular closure of Grade 3

 This study included 97 patients; 53 (54.6%) male and 44 (45.4%) female. The age of the patients ranged from 3 to 15 years (mean; 6.86± 5 years).VPV closure was Grade 4 in 71 patients (73%) and Grade 3 in 26 patients (27%). The pattern of closure was coronal in 49 (50.5%) patients and circular in 48 (49.5%) patients (Figs 1–4). 

The mean age of patients with coronal closure was 6.5 ± 3.87 years, and 7.2 ± 6 years for circular closure, with no significant difference between the groups (t=0.6842, P=0.4955) (Table.1). Patients with circular closure included 24 females and 24 males, while 32 male and 17 female patients had coronal closure. The difference in proportion of male and female patients did not differ between types of closure (P=0.127, Chi-square = 2.328) (Table.1). No infection, injury or bleeding was encountered during nasoendoscopy. The nasoendoscopy procedure itself could cause slight discomfort, although local anesthesia was provided.

**Table 1:** Age and gender of different velopharyngeal (VP) closure pattern

**VP closure**	**Coronal**	**Circular**	**Test**	**VP** ** closure**
Number (percent)	48 (49.5%)	49 (50.5 %)		
mean age	6.5 ± 3.87	7.2 ± 6	t = 0.6842	0.4955 NS
Sex				
Female	24	32	Chi-square = 2.328	0.127 NS
Male	24	17		

NS non-significant

## Discussion

The patterns of VPV closure are categorized into four closure types: coronal closure, sagittal closure, circular closure, and circular closure with Passavant’s ridge. The purpose of the present study was to explore the possible differences in VPV closure during speech production in apparently normal Arabic-speaking subjects.

In the current study, coronal closure was reported in 50.5% of children, followed by the circular pattern in 49.5%. The second important finding was the absence of the other two types of closure (sagittal closure and circular closure with Passavant’s ridge) among our patients.Witzel and Posnick similarly used nasopharyngoscopy and found that the predominant pattern of closure was coronal (68%), followed by the circular (23%), circular with a Passavant's ridge (5%), and finally sagittal (4%) (5). In contrast, Manochiopinig observed that circular closure is the most common type (6).

The absence of sagittal closure in this survey is of particular importance, as it raises doubt on the value of the surgical technique that uses lateral pharyngeal wall movement (sagittal closure) when choosing a surgical technique (as pharyngeal flaps with its modification) in the correction of the incompetence of VPV. Therefore, this correction will be static (or mechanical) by occluding the VPV gap, rather than dynamic (gaining benefit from the lateral pharyngeal walls).

However, there is also a dynamic element in pharyngeal flap surgery, as there are some types of lateral pharyngeal movement in the coronal and circular closure. In other words, judging the pattern of VPV closure depends on the predominant movement. In coronal closure, the velar movement is predominant, but this does not exclude the presence of sluggish lateral pharyngeal walls, while in circular closure, both the velum and lateral pharyngeal walls move in nearly equal degrees. Our observation of the absence of simultaneous lateral pharyngeal wall movement by flexible fiberoptic nasopharyngoscope in normal Arabic-speaking children is consistent with previous studies in other parts of the world using different speaking languages (9,10). However, it was suggested that it is difficult to demonstrate lateral pharyngeal walls using a flexible nasopharyngoscope, whereas this could be detected using another instrument, such as videofluoroscopy (9).

Manochiopinig et al found that some subjects might not exhibit their normal habitual behavior or may try too hard to achieve their best performance; however (6), this could not be concluded in our study as our cases were children.

It is thought that the Passavant’s ridge projects from the posterior pharyngeal wall into the pharynx during speech. This occurs as a result of the contraction of specific fibers of the superior pharyngeal constrictor muscles (6). Such a Passavant’s ridge is difficult to see through nasoendoscopy during complete or nearly complete VPV closure because its action clearly appears with a VPV closure and is usually located below the area of closure. Our findings also agreed with other studies in which the Passavant’s ridge is a rare phenomenon of the normal nasopharynx (4,6).

It is thought that the subject can change pattern of VPV closure by learning, in addition to anatomical variability including cleft palate (4). Therefore, it is possible to increase lateral wall motion by speech therapy prior to surgery.

The current study reported Grade 3 VPV closure in 27% of children with normal speech. This could be attributed to the effect of the adenoid and explains the previously reported post- adenotonsillectomy velopharyngeal insufficiency (VPI) (11). It also highlights the importance of preoperative VPV closure evaluation in children scheduled for adenotonsillectomy and including incompetent preoperative closure as one of the risk factors of post-adenotonsillectomy VPI.

A limitation in the current study is the use of only one technique (flexible nasopharyngoscope) in our evaluation of the VPV, because some subjects might not exhibit their normal habitual behavior or may try too hard to achieve their best performance. Nasoendoscopy requires close cooperation among the subject, the phoniatrician and the surgeon (9), which may be difficult in children; however, these children are the main target for cleft or VPI repair.

Further studies are recommended using several techniques, especially video-fluoroscopy and studying the pattern of VPV closure in normal or VPI patients before and after speech therapy to see if there is change in pattern or enforcing the lateral pharyngeal movement with speech therapy. In addition, short- and long-term flexible nasofibroscopic examination of patients after adenotonsillectomy will need further research to identify if there is a difference between preoperative and post-operative patterns and grades of VPV closure.

## Conclusion

Coronal and circular closures of the VPV are the main types of VPV in Arabic-speaking children, with a small insignificant superiority for the coronal closure. Neither the sagittal pattern nor circular with Passavant’s ridge was detected, raising the need to revise the methods of repair for persistent VPI. Moreover, the effect of speech therapy on lateral pharyngeal wall motion enhancement needs to be investigated.
